# Intensive training programme improves handwriting in a community cohort of people with Parkinson’s disease

**DOI:** 10.1007/s11845-023-03404-8

**Published:** 2023-05-30

**Authors:** Lucy M. Collins, Rachel Roberts, Hannah Cleary, James Diskin, Donna Kitt, Ingrid Van Bommel-Rutgers, Bouwien C. M. Smits-Engelsman, Erin K. Crowley, Aideen M. Sullivan

**Affiliations:** 1 Department of Anatomy and Neuroscience, University College Cork, Cork, Ireland; 2 Pharmaceutical Care Research Group, School of Pharmacy, University College Cork, Cork, Ireland; 3 Corrib Physiotherapy, Claregalway, Co, Galway, Ireland; 4 University for Professionals Avans+, Breda, The Netherlands; 5 Department of Health and Rehabilitation Sciences, University of Cape Town, Cape Town, South Africa; 6 Physical Activity, Sport and Recreation, Faculty Health Sciences, North-West University, Potchefstroom, South Africa

**Keywords:** e-Health, Handwriting, Micrographia, Parkinson’s disease, Physiotherapy

## Abstract

**Background:**

People with Parkinson’s disease (PwP) often report problems with their handwriting before they receive a formal diagnosis. Many PwP suffer from deteriorating handwriting throughout their illness, which has detrimental effects on many aspects of their quality of life.

**Aims:**

To assess a 6-week online training programme aimed at improving handwriting of PwP.

**Methods:**

Handwriting samples from a community-based cohort of PwP (*n* = 48) were analysed using systematic detection of writing problems (SOS-PD) by two independent raters, before and after a 6-week remotely monitored physiotherapy-led training programme. Inter-rater variability on multiple measures of handwriting quality was analysed. The handwriting data was analysed using pre-/post-design in the same individuals. Multiple aspects of the handwriting samples were assessed, including writing fluency, transitions between letters, regularity in letter size, word spacing, and straightness of lines.

**Results:**

Analysis of inter-rater reliability showed high agreement for total handwriting scores and letter size, as well as speed and legibility scores, whereas there were mixed levels of inter-rater reliability for other handwriting measures. Overall handwriting quality (*p* = 0.001) and legibility (*p* = 0.009) significantly improved, while letter size (*p* = 0.012), fluency (*p* = 0.001), regularity of letter size (*p* = 0.009), and straightness of lines (*p* = 0.036) were also enhanced.

**Conclusions:**

The results of this study show that this 6-week intensive remotely-monitored physiotherapy-led handwriting programme improved handwriting in PwP. This is the first study of its kind to use this tool remotely, and it demonstrated that the SOS-PD is reliable for measuring handwriting in PwP.

## Introduction

Parkinson’s disease (PD) is a debilitating chronic neurodegenerative disease, which is estimated to affect almost 10 million people worldwide [1]. The cardinal movement impairments are tremor, hypokinesia, rigidity, and bradykinesia. These impairments manifest as subtle but significant problems for people with Parkinson’s (PwP). Many of the less apparent symptoms appear before the onset of the cardinal motor symptoms [2–4]. These prodromal features include loss of sense of smell [5, 6], gastrointestinal problems [7, 8], and fine motor problems such as micrographia [5, 9]. Micrographia is defined as abnormally small or cramped handwriting [9], which often presents in early PD and gradually worsens with disease progression [5, 9]. This problem affects most PwP, at all stages of disease onset and progression [10–12]. Although micrographia in PwP inherently refers to handwriting size, it has become evident that PD also has detrimental effects on writing velocity, fluency, and acceleration [11]. Micrographia reduces patients’ overall quality of life in subtle yet noticeably debilitating ways [10, 11]. Variable improvements in handwriting have been reported following treatment with standard PD medications [13]. Therefore, adjuncts such as physiotherapy are commonly used, with the goal of preserving and improving motor symptoms such as micrographia.

The objective of this study was to assess the impact, using the Systematische Opsporing Schrijfproblemen (SOS-PD; systematic detection of writing problems for PwP) as an analytical tool, of a task-oriented remote handwriting training programme that was implemented by Corrib Physiotherapy Centre [14, 15]. The reliability and efficacy of the SOS-PD handwriting tool have been previously shown in PwP [14], in which the SOS-PD test results correlated with other analyses, such as the Purdue Pegboard and Manual Ability Measure (MAM-16) of fine motor skill performance. Various other experimental tools have also been used to systematically examine handwriting. For example, electronic tablets with paired stylus pens have been used to assess the dynamic features of handwriting in PwP [16], demonstrating potential to assess subjects’ handwriting and to assist clinicians in the early diagnosis of PD. Similarly, pressure-sensitive electronic tablets have also been used to assess handwriting competency in PwP; these allow enhanced accuracy and more thorough assessment of handwriting than paper- and pen-based assessments [17]. There is a need for a simple accessible tool to improve handwriting and thereby improve individuals’ quality of life at all stages of PD, especially for individuals with early-onset PD who are still in employment.

## Methods

**Fig. 1:**
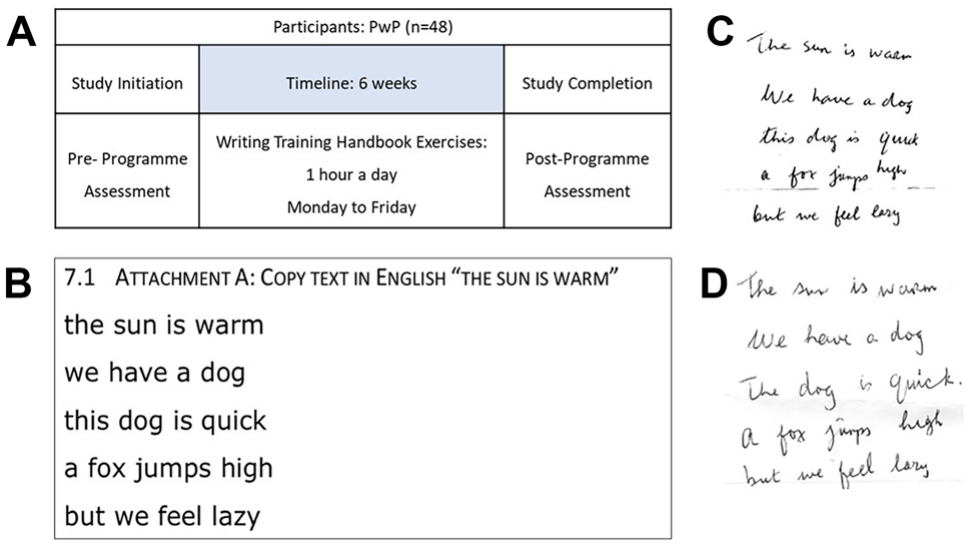
**A** Outline of the study design, **B** sample text to be copied in the assessment, **C** sample of pre-programme handwriting from a study participant, and **D** sample of post-programme handwriting from the same study participant

Ethical approval was granted through the University College Cork (UCC) Social Research Ethics Committee (SREC), log number 2021–070. The study was performed in accordance with the ethical standards as laid down in the 1964 Declaration of Helsinki and its later amendments or comparable ethical standards. In total, 48 participants were recruited to the handwriting training programme by Corrib Physiotherapy, Co. Galway, Ireland. The participants were not pre-selected and represented a broad range of disease duration. Each participant was given a workbook with exercises to complete each day for 6 weeks and had access to a video demonstrating each exercise. The training for the intervention was carried out by Corrib Physiotherapy and followed the same protocol as previously published [18]. The data was analysed retrospectively by the team in UCC, who were blinded to the participants’ descriptive/identifiable information. The raters were fully trained in the SOS scoring system. The pre- and post-programme assessments involved copying a set of sentences (Fig. 1A, B). An example of pre-programme handwriting text from a participant is shown in (Fig. 1C). A post-programme handwriting sample by the same participant is shown in Fig. 1D. The SOS-PD consists of an instruction manual, a paper copy of the text to be written by participants, a scoring form, and a measuring template to allow for objective scoring of the handwriting. Over the 6 weeks between the two assessments, participants spent 1 h per day for 5 days per week completing exercises in the handwriting training handbook supplied to them by Corrib Physiotherapy. The following aspects of the handwriting samples were assessed, based on criteria described in the SOS-PD manual: writing fluency, transitions between letters, regularity in letter size, word spacing, and straightness of lines. Average handwriting size in millimetre and copying speed were also assessed but did not contribute to the overall SOS-PD score. A higher total SOS-PD score indicated poorer quality of handwriting. The sentences were scored using the SOS-PD. Speed was assessed by measuring the number of characters copied by the participant in 5 min. Handwriting texts were also scored based on the number of illegible and legible words, to calculate the percentage legibility of the copied text. Additionally, participants were scored on several observational parameters, carried out by a physiotherapist working with Corrib Physiotherapy. These parameters included whether a tremor was observed in the writing hand, whether a tremor was observed in the paper-holding hand, if upper limb freezing was observed, if the patient always wrote with interruptions between letters, and whether the handwriting style changed over the course of the training period, i.e., cursive versus non-cursive writing.

Statistical analysis of the data was performed using IBM SPSS Statistics version 27. The data was not normally distributed, therefore, non-parametric tests were carried out. The McNemar test and Wilcoxon signed rank tests were used to compare the pre- and post-programme data, to determine whether there were statistically significant differences between any of the pre- and post-programme measures. Two raters (rater 1 and rater 2) were blinded to each other’s results. Inter-rater reliability (IRR) for the SOS-PD scoring was determined using the pre- and post-programme handwriting samples. IRR categorical variables were assessed by Cohen’s Kappa and interclass correlation coefficient (ICC) for IRR continuous variables. Cohen’s interpretation of the Kappa result was used as follows: values ≤ 0 indicate no agreement, 0.01–0.20 no to slight agreement, 0.21–0.40 fair, 0.41–0.60 moderate, 0.61–0.80 substantial, and 0.81–1.00 almost perfect agreement. As levels of agreement between the independent raters were sufficient, data from both raters were averaged for each variable to achieve a combined dataset which was used for analysing the outcome variables before and after the intervention. Clustered bar charts were then generated to represent the data. The significance level was set at *p* < 0.05 for all tests.

## Results

### Participant demographics

A total of 48 participants took part in this study. Sixteen (33%) of the participants did not continue with the follow-up assessment. In this cohort of participants who did not complete the assessment, the majority (75%) were male and right-handed (93.75%), with a mean age of 71.00 ± 7.32 years. In the remaining cohort of 32 patients who completed the follow-up assessments, the majority (90.6%) of the participants were right-handed, and 53.12% were female. The average age of this completing group was 70.75 ± 6.89 years and the average PD duration was 9.19 ± 6.06 years (Table 1). Observed tremor in the writing hand was found to be decreased in the post-programme assessment compared to the pre-programme assessment (*p* = 0.027), whereas observations of tremor in the non-writing hand (*p* = 0.687) and upper limb freezing (*p* = 0.178) were not significantly different between the pre- and post-programme assessments. There were no differences in the timing of medication intake relative to the timing of the pre- and post-programme assessments in individual participants. All assessments were performed soon after the participants had taken their PD medication. The most common medications used by the group of 32 participants who completed the study were Sinemet (carbidopa and levodopa) (*n* = 12), Stalevo (levodopa, carbidopa, and entacapone) (*n* = 6), Sinemet Plus (carbidopa and levodopa) (*n* = 5), and Madopar (levodopa and benserazide) (*n* = 4). Other participants were taking amantadine (*n* = 1), Requip (ropinirole) (*n* = 1), Duodopa pump (carbidopa and levodopa) (*n* = 1), Azilect (rasagiline) (*n* = 1), and Neupro patch (*n* = 1).

**Table 1:** Participants’ demographics. In cases of normal distribution and equality of variances, mean ± standard deviation is shown

Demographic	All participants (*N* = 48)	Completing participants (*N* = 32)	Drop-out participants (*N* = 16)
Gender (M/F)	27/21	15/17	12/4
Handedness (R/L)	44/4	29/3	15/1
Age (years)	70.83 ± 6.96	70.75 ± 6.89	71.00 ± 7.32
Disease duration (years)	10.02 ± 6.30	9.19 ± 6.06	11.69 ± 6.63
Pre-programme cursive handwriting (yes/no)	16/32	11/21	5/11
Post-programme cursive handwriting (yes/no)	14/18	14/18	NA

### Analysis of inter-rater reliability

IRR was used to assess the levels of agreement between individual raters. The agreement between rates on the overall SOS-PD score, size, speed, legible words count, illegible words count, and percentage legibility was high (Table 2). However, the agreement on some of the individual SOS-PD items was low, specifically fluency, regularity, word spacing, and straightness of line. There was not a statistically significant level of agreement between the raters on transitions between letters, fluency, and straightness of line (Table 2). There was moderate agreement between the raters regarding whether the writing was considered to be cursive or not (Table 2).

**Table 2:** Analysis of inter-rater reliability. Measurement units: SOS-PD speed (letters written in 5 min); IRR (Cohen’s Kappa) (items 1–6). *ICC (interclass correlation coefficient) value (items 7–12)

Inter-rater reliability						
	Pre-programme assessment			Post-programme assessment		
Item	IRR/ICC	*p*-value	Agreement	IRR/ICC	*p*-value	Agreement
1. Cursive (0–1)	0.646*	< 0.001	Moderate	0.685	< 0.001	Moderate
2. Fluency (0–2)	0.229	0.074	Minimal	0.265	0.035	Minimal
3. Transitions (0–2)	0.069	0.686	Minimal	0.175	0.220	Minimal
4. Regularity (0–2)	0.313	0.034	Minimal	0.228	0.106	Minimal
5. Word spacing (0–2)	0.277	0.039	Minimal	0.388	0.001	Minimal
6. Straightness of line (0–2)	0.265	0.072	Minimal	0.483	0.002	Weak
7. Total SOS-PD score (0–10)	0.544*	< 0.001	Moderate	0.598*	< 0.001	Moderate
8. Size (mm)	0.905*	< 0.001	Excellent	0.868*	< 0.001	Good
9. Speed (letters in 5 min)	0.987*	< 0.001	Excellent	0.975*	< 0.001	Excellent
10. Legible words (count)	0.884*	< 0.001	Good	0.971*	< 0.001	Excellent
11. Illegible words (count)	0.861*	< 0.001	Good	0.918*	< 0.001	Excellent
12. % legible	0.841*	< 0.001	Good	0.908*	< 0.001	Excellent

IRR categorical variables were assessed by Cohen’s Kappa and interclass correlation coefficient (ICC) for continuous variables (*n* = 32)

*p < 0.05; **p < 0.01; ***p < 0.001

### Pre- and post-programme assessment of participants’ handwriting quality

**Fig. 2:**
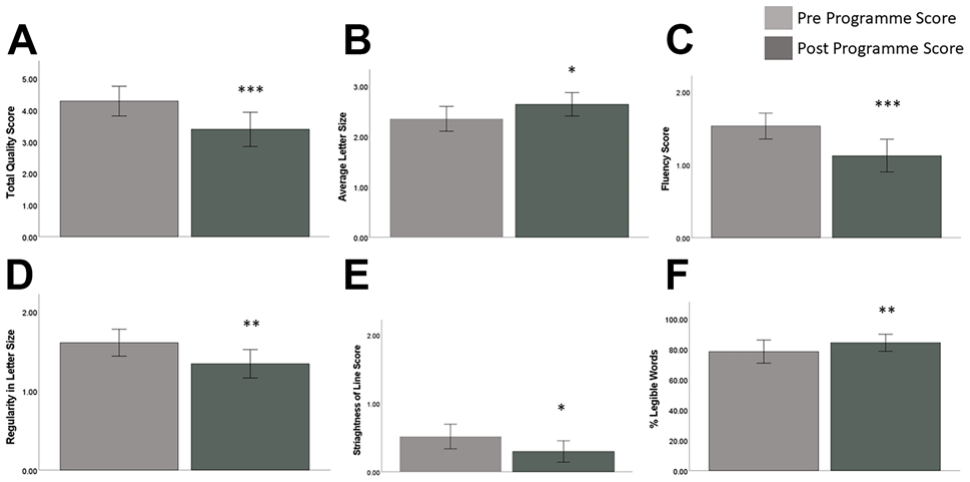
SOS-PD handwriting quality scores in participants on pre- and post-programme assessments. **A** Total SOS-PD score (0–10), **B** letter size, **C** fluency score, **D** regularity of letter size, **E** straightness of line, **F** percentage legible words. *p < 0.05; **p < 0.01; ***p < 0.001 vs pre-programme assessment score; McNemar test and the Wilcoxon signed rank test (*n* = 32)

Assessment of handwriting style at the pre- and post-programme stages showed that there was no significant change in the overall style of the participants’ handwriting (i.e., whether they used cursive or non-cursive handwriting) over the 6-week training programme (*p* = 0.506; Table 3). After the 6-week training period, participants’ overall SOS-PD score had decreased, showing that the overall quality of their handwriting had improved (*p* < 0.001; Table 3 and Fig. 2A). Furthermore, their writing size had increased after the 6-week training programme (*p* = 0.012; Table 3 and Fig. 2B), but their handwriting speed was not significantly different between pre- and post-programme stages (*p* = 0.405; Table 3). Further analysis revealed that at the post-programme stage, participants’ writing fluency (*p* < 0.001; Table 3 and Fig. 2C), regularity of letter height (*p* = 0.009; Table 3 and Fig. 2D), and straightness of sentences (*p* = 0.036; Table 3 and Fig. 2E) were all significantly improved compared to the pre-programme assessment. There were no significant differences in transitions between letters (*p* = 0.187; Table 3) and spacing between words (*p* = 0.317; Table 3) at the pre- and post-programme stages. Regarding the legibility of the text, the number of illegible words significantly decreased after the 6-week training programme (*p* = 0.009; Table 3), but there was no change in the number of legible words (*p* = 0.992; Table 3). However, overall percentage legibility improved following training (*p* = 0.008; Table 3 and Fig. 2F), meaning that in general, the participants’ handwriting was easier to read after they had completed the training programme.

**Table 3:** Handwriting scoring based on SOS-PD

	Pre-programme assessment			Post-programme assessment		
Item (unit)	Rater 1	Rater 2		Rater 1	Rater 2	*p*-values
Cursive	1.66	1.69		1.56	1.53	0.506
Fluency (0–2)	1.41	1.66		1.16	1.09	< 0.001
Transitions (0–2)	1.64	0.64		1.43	0.53	0.187
Regularity (0–2)	1.47	1.75		1.25	1.44	0.009
Word spacing (0–2)	0.09	0.31		0.09	0.22	0.317
Straightness of line (0–2)	0.63	0.41		0.34	0.25	0.036
Total SOS-PD score (0–10)	4.16	4.41		3.47	3.31	< 0.001
Size (mm)	2.25	2.45		2.56	2.72	0.012
Speed (letters in 5 min)	333.66	338.28		333.72	335.66	0.405
Legible words (count)	52.44	60.31		54.03	61.97	0.992
Illegible words (count)	19.22	11.97		15.31	7.63	0.009
% legible words	73.75	83.11		78.66	89.93	0.008

Measurement units: total SOS-PD score (0–10); SOS-PD speed (letters written in 5 min) (*n* = 32)

## Discussion

Micrographia is a common motor symptom in PwP and can have profound negative impacts on quality of life. This study assessed the impact of a 6-week remote training programme on handwriting of PwP. It was the first of its kind in Ireland and was conducted through a community-based online physiotherapy clinic. In our study, two independent raters marked the handwriting assessments, and we subsequently assessed the reproducibility between these two raters, who had been blinded to each other’s scoring. Our analysis showed high inter-rater reliability between these raters for total SOS-PD score, size, and speed, as well as the legibility scores. For the internal items, there were variable findings, with fluency, transitions, regularity, word spacing, and straightness of lines showing moderate to low agreement, in line with previous reported assessments [14], suggesting that some of the SOS-PD items may be redundant in these types of studies in PwP.

The participants were not pre-selected and represented a broad range of disease duration. Overall, results from the data analysis suggest that, following completion of the 6-week programme, participants’ handwriting improved. There was a significant decrease in the total SOS-PD score, which is reflective of an increase in handwriting quality and legibility. Further analysis of the handwriting scores showed that size, fluency, regularity of letter size, and straightness had all improved after training. These findings are important, as fluency and regularity of letter size have previously been found to be the most affected aspects of handwriting in PwP [14, 15, 19]. One previous study reported that size and progressive letter height in a sentence were the main handwriting problems exhibited by PwP [14]. We found no significant changes in the speed of handwriting, in transition between letters, or in space between words, between pre- and post-programme assessments. This is consistent with a previous study that used the SOS-PD tool in PwP and also reported no significant changes in these items [14]. Furthermore, increasing handwriting speed was not a goal of the training programme. The focus was on increasing the size of each letter and working on fluency. It is interesting that, in our study, there were improvements in several SOS-PD items, thus resulting in an overall enhancement in the legibility of the text, without slower letter production. This data is supported by our additional independent assessments of handwriting legibility, where a significant decrease in illegible words was documented, along with a corresponding increase in total percentage legibility. These items were a novel assessment to supplement the SOS-PD and have not been carried out in PwP to date.

All patients continued to take their regular medications throughout the study, as medication timings are imperative to symptom management in PD. Regarding our study protocol, we did not observe a significant change in the timing of medication intake between the pre- and post-programme assessments. Freezing and tremor in participants' non-writing hands were not observed to change over the course of the programme. However, participants were noted to have significantly less tremor in their writing hand at the post-programme assessment. Although it is noteworthy that this was an observed tremor, we cautiously conclude that tremor does not have a significant impact on handwriting as reflected in participant’s scores. Tremor can be a bothersome symptom for PwP. Based on the findings of this study, PwP can be reassured that although they have a tremor, it should not impact upon their handwriting, as the scores do not show any effect of tremor. It has been suggested that micrographia is a component of bradykinesia, as the two symptoms are correlated [14], and indeed tremor may not affect speed or size of handwriting. Interestingly, it was observed that in the Post-Programme assessments, the handwriting style of three of the participants changed from being cursive to non-cursive, upon completion of the programme. This may be reflective of increased control of fine motor movement, or that the participant was more attentive or cognisant of their handwriting style after completion of the training programme.

Participants were from a community setting and represented various stages of PD. The physiotherapists did not exclude people based on stage of disease or on disease duration. This is important as it means that even people who were considered to have severe PD were offered the opportunity to complete a training programme that targets handwriting issues, symptoms that are known to be important to them. There was a significant drop-out rate in the programme, which reflects the intensity of the programme being considered to be too high by some participants. This does not necessarily mean that those participants who dropped out did not benefit from the programme. They received a detailed workbook and information on the training programme, and so it is possible that they continued the programme at their own pace.

The observed improvements in handwriting in our study have potential to improve the quality of life for PwP. This training programme could relatively easily be implemented in the care of individuals with early-stage PD, potentially delaying or preventing the observed micrographia seen in many PwP, thereby preserving fine motor control and improving independence and quality of life. PwP frequently report frustration relating to micrographia, as it can impede their independence [11, 15]. Future studies could select individuals with earlier stages of PD to complete this handwriting programme, perhaps with modifications. Writing aids such as rulers, weighted pens, or different pen types, such as fountain pens, could be used in conjunction with the programme. It is relevant to consider whether the conditions of remote assessments used in our study may have affected the performance of participants. Here, participants performed the assessments in their own homes, in a relaxed and familiar setting. There may have been different outcomes if the assessments had been carried out in a more stressful clinical environment. It is important to note, however, that the influence of a ‘practice effect’ may have contributed to the observed differences in our study, by altering participants’ respective performances during the repeated assessments [20].

Our study lacked a control group, but previous studies using this training method had established the training protocol and found measurable differences in size and speed of handwriting when comparing PwP to a control group [14]. The SOS assessment has also been reported to confer improvements of up to 17% in size of handwriting in PwP [18]. In our study, we found 20% improvement in overall SOS scores, 8% change in the size, and 7.5% improvement in handwriting legibility. To note, 16 of the participants did not complete our study, due to the intensity of the training programme. These participants reported hand and muscle cramps, as well as fatigue. Future participants could be advised to include relaxation movements or timed breaks, to alleviate fatigue. The training programme was highly intensive, comprising of 1 h per day for 5 days, over 6 weeks. In future studies, participants could be stratified based on their disease stage, with a more intensive programme used for people with early-stage PD and a less intensive one for individuals with advanced PD, to tailor the handwriting programme to suit the needs of the individual. In another study, good retention of participants was achieved in a less intensive programme over a longer period of time [20]. The online nature of this training has potential to increase the accessibility of resource, for example, to those living in rural communities without access to physiotherapy clinics. Further flexibility could also be implemented in the programme; for example, individuals with PD who are working may choose to take the course online or to use recorded training videos.

## Data Availability

Data are available on Zenodo at this link: https://zenodo.org/record/7967603
